# British Cancer Research Meeting 2000

**DOI:** 10.1054/bjoc.2000.1434

**Published:** 2000-08-10


					British Cancer Research Meeting 2000
9-12 July 2000
Brighton, UK
1
British Journal of Cancer (2000)
(c) 2000 Cancer Research Campaign
doi: 10.1054/ bjoc.2000.1434, available online at http://www.idealibrary.com on

				

